# Construction of a cellulase hyper-expression system in *Trichoderma reesei *by promoter and enzyme engineering

**DOI:** 10.1186/1475-2859-11-21

**Published:** 2012-02-08

**Authors:** Gen Zou, Shaohua Shi, Yanping Jiang, Joost van den Brink, Ronald P de Vries, Ling Chen, Jun Zhang, Liang Ma, Chengshu Wang, Zhihua Zhou

**Affiliations:** 1 Key Laboratory of Synthetic Biology, Institute of Plant Physiology and Ecology, Shanghai Institutes for Biological Sciences, Chinese Academy of Sciences, Shanghai 200032, China; 2 Fungal Physiology, CBS-KNAW Fungal Biodiversity Centre, Uppsalalaan 8, 3584 CT Utrecht, The Netherlands

**Keywords:** Fusion protein, Site-specific mutagenesis, Heterologous expression, Transcriptional regulation, Cellulase

## Abstract

**Background:**

*Trichoderma reesei *is the preferred organism for producing industrial cellulases. However, a more efficient heterologous expression system for enzymes from different organism is needed to further improve its cellulase mixture. The strong *cbh1 *promoter of *T. reesei *is frequently used in heterologous expression, however, the carbon catabolite repressor CREI may reduce its strength by binding to the *cbh1 *promoter at several binding sites. Another crucial point to enhance the production of heterologous enzymes is the stability of recombinant mRNA and the prevention of protein degradation within the endoplasmic reticulum, especially for the bacteria originated enzymes.

In this study, the CREI binding sites within the *cbh1 *promoter were replaced with the binding sites of transcription activator ACEII and the HAP2/3/5 complex to improve the promoter efficiency. To further improve heterologous expression efficiency of bacterial genes within *T. reesei*, a flexible polyglycine linker and a rigid α-helix linker were tested in the construction of fusion genes between *cbh1 *from *T. reesei *and *e1*, encoding an endoglucanase from *Acidothermus cellulolyticus*.

**Results:**

The modified promoter resulted in an increased expression level of the green fluorescent protein reporter by 5.5-fold in inducing culture medium and 7.4-fold in repressing culture medium. The fusion genes of *cbh1 *and *e1 *were successfully expressed in *T. reesei *under the control of promoter pcbh1m2. The higher enzyme activities and thermostability of the fusion protein with rigid linker indicated that the rigid linker might be more suitable for the heterologous expression system in *T. reesei*. Compared to the parent strain RC30-8, the FPase and CMCase activities of the secreted enzyme mixture from the corresponding transformant R1 with the rigid linker increased by 39% and 30% at 60°C, respectively, and the reduced sugar concentration in the hydrolysate of pretreated corn stover (PCS) was dramatically increased by 40% at 55°C and 169% at 60°C when its enzyme mixture was used in the hydrolysis.

**Conclusions:**

This study shows that optimizations of the promoter and linker for hybrid genes can dramatically improve the efficiency of heterologous expression of cellulase genes in *T. reesei*.

## Background

Limited fossil resources, growing economies and an everlasting burden on our environment have caused an increasing interest for alternative resources to produce fuels and chemicals. Efficient conversion of lignocellulosic biomasses, the largest renewable resource on earth, requires cost-effective enzyme systems to degrade the polysaccharides to monomeric compounds [1]. The current enzyme mixtures for the bioconversion of lignocellulose are not sufficiently efficient for an economic viable biorefinery of plant biomasses. The filamentous fungus *Trichoderma reesei *(teleomorph *Hypocrea jecorina*) [2] is by far the preferred organism for production of cellulases within industry [3,4]. To enable efficient degradation of cellulose, the co-operation of at least three types of enzymes is required: cellobiohydrolases, endoglucanases and β-glucosidases. Because cellobiohydrolase I (CBHI, EC 3.2.1.91) and cellobiohydrolase II (CBHII, EC 3.2.1.91) comprise nearly 85% of the total secreted proteins of *T. reesei *[5-7], the current commercial cellulase mixture used for biomass hydrolysis requires a cocktail consisting of cellulases produced by *T. reesei*, and β-glucosidase and new endoglucanases from other fungi or bacteria [8]. Another limitation of the cellulases produced by *T. reesei *is their relatively low thermostability [5,9]. Higher reaction temperatures associated with thermostable cellulases during the hydrolytic process may radically reduce substrate viscosity leading to higher reaction velocities and better substrate conversion at lower energy consumption [10-12]. To improve the cellulase mixture of *T. reesei *in its composition and thermostability, homologous or heterologous expression of cellulase genes other than *cbh1 *and *cbh2 *is necessary in *T. reesei*.

The promoter *cbh1 *of *T. reesei *is known to be a strong inducible promoter, and is therefore commonly used to construct high-efficient heterologous expression vectors in *T. reesei *and other fungi [13,14]. However, three putative carbon catabolite repressor binding sites are present in the region from -685 to -724 nt of the *cbh1 *promoter. They are considered to reduce transcripts of *cbh1 *when glucose is present in the fermentation medium [15,16]. The deletion of these repressor binding sites and introduction of multi-copy activator binding sites in *cbh1 *promoter not only eliminated the glucose repression effect, but also increased promoter activity and production levels of heterologous proteins in *T. reesei *Rut-C30 [17]. Except for the main repressor protein CREI, many other transcription factors (TF) of *T. reesei *have been identified, such as the repressor ACEI, and the positive regulators XYRI, ACEII and the HAP2/3/5 complex [18]. Within this study, we are testing the hypothesis that replacing the negative regulator binding sites of the *cbh1 *promoter for positive regulator binding sites may further improve the expression level of heterologous genes.

Even though bacteria contain cellulases with interesting properties, heterologous expression in *T. reesei *of genes originated from bacteria is often causing problems [19-21]. One of the most obvious reasons for low expression levels might be the degradation of the heterologous cellulases by the abundant proteases produced in the fungal host [22,23]. This issue can be solved by stabilizing the recombinant protein, for instance by creating a fusion with a native protein. The fusion protein will serve as a carrier to facilitate the translocation of the foreign protein in the secretory pathway and, thereby, protect the heterologous part from degradation [14,24].

The endoglucanase E1 (EC 3.2.1.4), secreted by thermophilic bacterium *Acidothermus cellulolyticus*, will be used as a case-study to test a novel heterologous expression system within *T. reesei*. The distinctive characteristics of this enzyme were shown to be of high potential for industry [21,25]. Besides its robustness due to extreme thermostability of endoglucanase E1, it also has a striking synergism with cellulases of *T. reesei *at high temperature [26]. Furthermore, the heterologous expression of *e1 *in corn has shown to facilitate conversion of pretreated corn stover (PCS) into glucose [21]. The catalytic domain of *e1 *was also successfully expressed in *T. reesei *when fused with the catalytic domain of *cbh1*, resulting in a 30% increase of PCS hydrolysis efficiency at 55°C [13]. However, whether the fused protein could improve thermostability of the complete cellulase complex from *T. reesei *is still unknown.

In this study, the three CREI binding sites in *cbh1 *promoter were replaced by the binding sites of positive regulator ACEII or HAP2/3/5 complex, and the efficiency of the modified promoters were quantified using the enhanced green fluorescent protein (EGFP) as reporter. A flexible neutral polyglycine linker and a rigid α-helix linker were used to fuse *cbh1 *from *T. reesei *and *e1 *from *A. cellulolyticus*. In order to make sure whether the fusion protein would result in an increase of CBHI thermostability, the intact *cbh1 *gene instead of the core region was used in the fusion gene. This expression system, with the novel *cbh1 *promoter and the different linkers between the intact CBHI and endoglucanase E1, was characterized for its cellulase activity, thermostability and hydrolytic efficiency against PCS at 50°C-75°C.

## Results and discussion

### Replacing the CREI binding sites for transcription activator binding sites in the promoter *cbh1 *increased its ability to express heterologous genes

**Figure 1:**
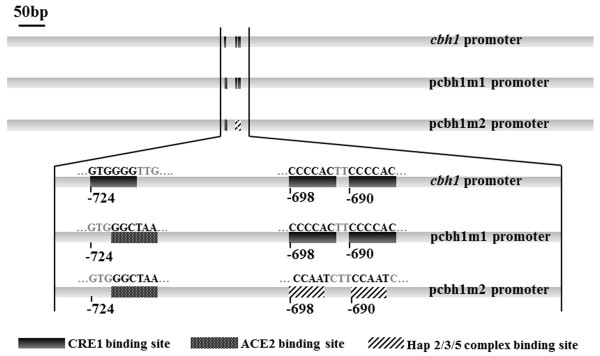
**Schematic structure of *cbh1 *promoter and its mutants**. There are three CREI binding sites located at -690, -698 and -724 in wild type *cbh1 *promoter. An ACEII binding site was replaced the CREI binding site at -724 in promoter pcbh1m1. Based on pcbh1m1, HAP2/3/5 complex binding sites were substituted for the remaining two CREI binding sites in promoter pcbh1m2.

Expression of *cbh1 *is dramatically decreased when repressor CREI is bound to its promoter, especially in culture media containing glucose. The deletion of the three CREI binding sites and the repetition of multi-copy regions with activator binding sites resulted in an increase of *cbh1 *promoter efficiency and thus a higher expression level of heterologous proteins in *T. reesei *[17]. May the *cbh1 *repressor binding sites replaced by its activators recognizing-sites also improve its activity? In this study, two newly engineered *cbh1 *promoters were obtained by site specific mutagenesis: pcbh1m1, in which -724 CREI motif was changed to the binding site of transcription factor ACEII (5'-GGCTAA-3'), and pcbh1m2, in which the two other CREI motifs at -698 and -690 within the pcbh1m1 promoter were changed to the binding site of the HAP2/3/5 protein complex (5'-CCAAT-3') (Figure 1). The specific-site mutageneses were confirmed by sequencing. In order to compare the strength of the wild type *cbh1 *promoter and its two mutants, the enhanced green fluorescent protein reporter gene (*egfp*) was placed behind each promoter. This resulted in three expression vectors pDHt/sk-pcbh1, pDHt/sk-pcbh1m1 and pDHt/sk-pcbh1m2.

**Figure 2:**
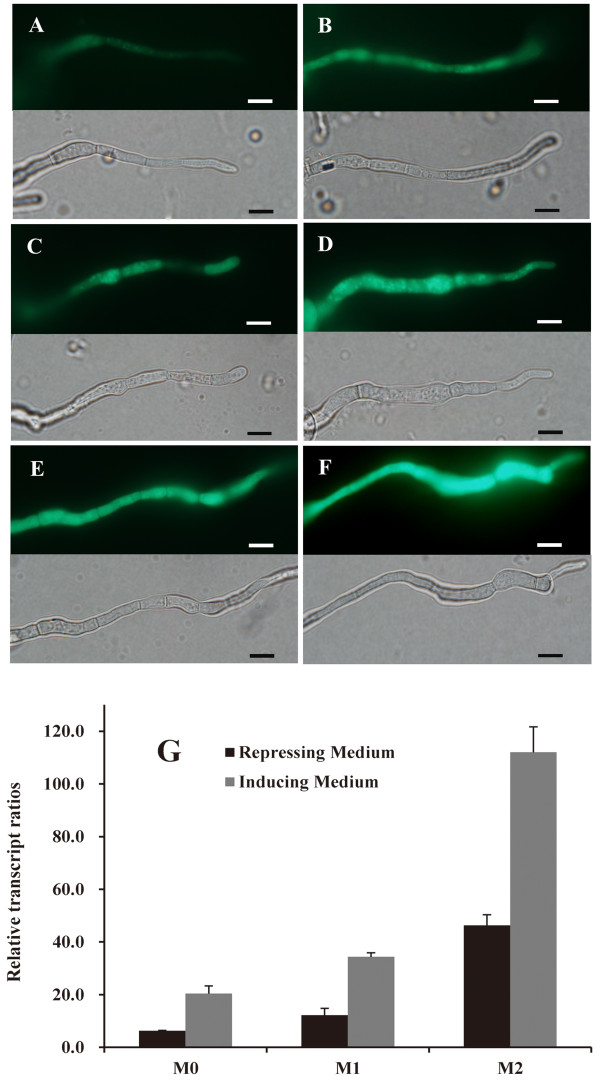
**Qualitative and quantitative evaluation of promoter strength via the expression level of *egfp *reporter gene**. Fluorescence microscopy of EGFP in *Trichoderma *hyphae (A - F). Transformant M0 (A and B) was under control of the wild type *cbh1 *promoter, and M1 (C and D) and M2 (E and F) corresponded to the mutated promoters pcbh1m1 and pcbh1m2, respectively. The GFP channel is shown in the top part of each figure, while the lower part shows the bright field image. *T. reesei *hyphae under the repressing condition (2% glucose as repressor) were shown in A, C and E, while those under the inducing condition (3% cellulose and 2% wheat bran as inducer) were in B, D and F. Scale bar = 5 μm. Relative expression levels of *egfp *(G) were calculated in comparison with the expression of *act *encoding for actin. Error bars are representing the standard deviation between three independent measurements.

After transformation and three consecutive subcultures for genetic stability, five mitotically stable transformants with single-copy of the fused genes were selected from each transformation. M0, M1 and M2 represented the transformants with vectors pDHt/sk-pcbh1, pDHt/sk-pcbhm1 and pDHt/sk-pcbhm2, respectively (Figure 2). The promoter strengths of all selected transformants were assessed qualitatively by fluorescence microscopy. The mycelia of all transformants glowed with clear bright green fluorescence after 1 day of growth on inducing culture media, *i.e*. containing a mix of wheat bran and cellulose just as M0 shown in Figures 2B, D, F. In contrary, transformants with the wild *cbh1 *promoter radiated weak fluorescence during growth on repressing culture media (containing 2% glucose) (Figure 2A), while transformants with the modified promoter like M1 and M2 still showed bright fluorescence (Figure 2C, E). Although Rut-C30 has a truncated CREI [27], the CREI-mediated carbon catabolite repression appeared to be not completely abolished in its derivative RC30-8.

The promoter strengths of M0, M1 and M2 were further quantitatively by real-time (RT) PCR based on the expression level of *egfp *(Figure 2G). Relative to M0 grown on repressing medium, RT-PCR results showed that the expression level of *egfp *in transformant M1 was increased by 1.9- and 1.7-fold in inducing and repressing culture media, respectively (Figure 2G). These observations imply that the first mutagenesis, in which -724 motif of *cbh1 *promoter was replaced with the ACEII binding site, did increase the strength of the promoter. However, the increase was not large and was similar to the deletion of this CREI motif, as shown in the study of Liu *et al*. [17]. The replacements of the other two motifs at -698 and -690 with the binding site of the HAP2/3/5 protein complex did result in a significant increase of promoter strength. The *egfp *expression level showed 7.4- and 5.5-fold increase compared to M0 under the inducing and repressing condition, respectively (Figure 2G). The strength of the mutated promoter pcbh1m2 was much stronger compared to the mutated promoter ΔpC in which all of the three CREI binding sites were deleted, and was also stronger than the mutated promoter Δp4C, in which four copies of ACEII and HAP2/3/5 complex binding sites were inserted in promoter ΔpC [17].

The cellulose-induced cumulative effect of positive regulatory factors was clearly observed in our study. Even during growth on the repressing media, the pcbh1m2 promoter resulted in a stronger expression of *egfp *than the original *cbh1 *promoter during growth on inducing medium. Although only a few transcripts of genes are independent of CREI in *T. reesei*, carbon catabolite repression (CCR) involves interaction of many other transcription factors [28,29]. In addition to CREI, three other proteins, CREII, CREIII and CREIV, participate in CCR. Furthermore, glucokinase (GLKI) and hexokinase (HXKI) are also involved in CREI-mediated CCR [18]. The level of derepression in *Δglk1/Δhxk1 *strains was higher compared to the *Δcre1 *mutant Rut-C30 [18]. Consequently, straightforward deletion of CREI binding sites cannot abolish CCR. Our results reveal that replacing repressor binding sites within promoters with a variety of activator binding sites is a powerful tool to enhance expression levels of heterologous proteins.

### The linker design showed significant effects on the efficiency of heterologous expression of a bacterial cellulase gene in *T. reesei*

The modified promoter pcbhm2 together with the signal sequence of *cbh1 *was used for transforming *T. reesei *RC30-8 with the intact ORF or the catalytic domain of endocellulase E1 from bacterium *A. cellulolyticus*. Unfortunately, no corresponding protein products were detected in 23 positive transformants via SDS-PAGE or western blotting (data not shown). Expressing and synthesizing bacterial cellulase genes directly in fungi requires overcoming several severe obstacles, such as compatibility of codon bias for correct transcription, stability of the bacterial mRNA, and misfolding or proteolysis after translation [19,20,30]. The *e1 *mRNAs were detected in those transformants by reverse transcription PCR (data not shown), demonstrating that *e1 *or its catalytic domain was transcribed by *T. reesei*. Deductively, E1 was most likely misfolded and then proteolyzed by endoplasmic-reticulum-associated protein degradation (ERAD).

**Figure 3:**
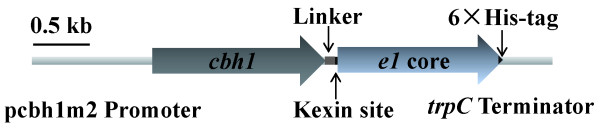
**Schematic structure of the fusion gene *tce1-fle *and *tce1-rig***. Some expressed fusion proteins would be cleaved at the kexin site (Lys-Arg) by the extracellular proteinases of *T. reesei*.

**Figure 4:**
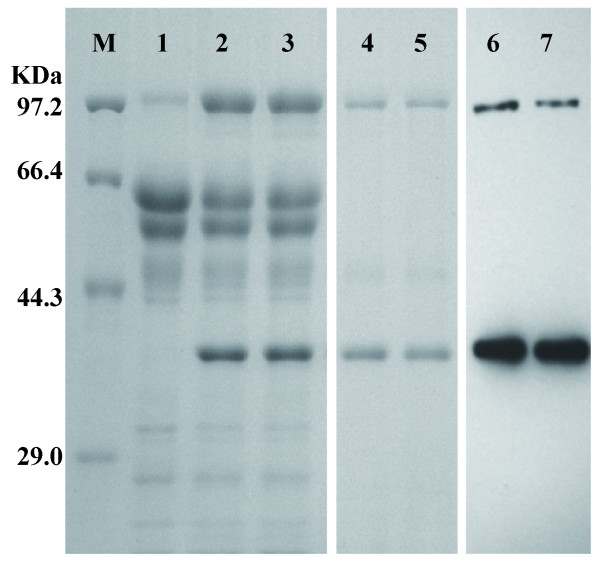
**SDS-PAGE and western blots of *T. reesei *RC30-8 and its transformants containing TCE1-FLE or TCE1-RIG**. Lane M, protein markers; lanes 1, 2 and 3, the total secreted proteins in the culture filtrate of wild type, transformant with TCE1-FLE and transformant with TCE1-RIG; lane 4 and 5, the purified TCE1-FLE and TCE1-RIG using Novagen Ni-NTA His•Bind^® ^Resin; lane 6 and 7, verification of TCE1-FLE and TCE1-RIG by Western blotting using an anti-His antibody. Unrelated lanes were removed for clarity.

Fusion of a heterologous gene with a native gene has been reported to stabilize the recombinant mRNA, facilitate translocation of the foreign protein in the secretory pathway, and avoid protein degradation [20]. To be able to express *e1 *in *T. reesei*, two types of linkers, a flexible neutral polyglycine linker (GGGGS)_4 _and a rigid α-helix linker (EAAAR)_4 _[31] were used to fuse the complete coding region of CBHI and the E1 catalytic domain. The two constructs, *i.e. tce1-fle *(with flexible neutral polyglycine linker) and *tce1-rig *(with rigid α-helix linker), were under control of the novel strong promoter pcbhm2 and contained a his-tag at the *e1 *catalytic domain (Figure 3). When transformed to *T. reesei *RC30-8, the corresponding fusion proteins and the cleaved E1 catalytic domain were detected in the extracellular enzyme mixture after growth on the inducing media containing wheat bran and cellulose for all positive transformants by SDS-PAGE and Western blotting (Figure 4). Two bands of approximately 97 and 40 kDa were detected on Western blots. The large band represented the complete fusion proteins TCE1-FLE or TCE1-RIG, while the smaller band represented the E1 catalytic domains. It means that a portion of the two linkers could be cleaved due to its kexin cleavage site (Lys-Arg) and thus E1 could be separated from the fusion proteins during the process of the protein secretion and purification. This strategy showed to be an effective way to protect the bacterial endoglucanase E1 from protein degradation within the endoplasmic reticulum due to misfolding [31-33].

**Figure 5:**
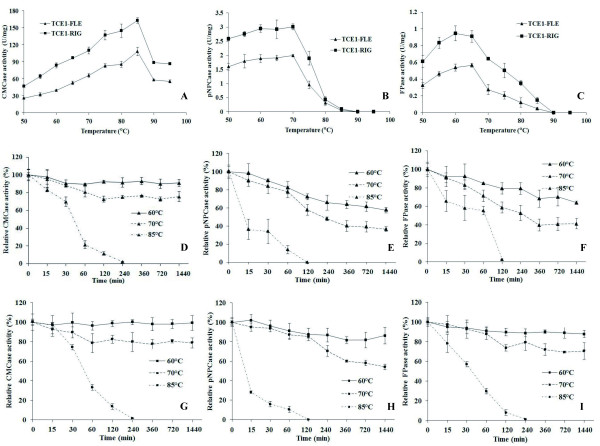
**Cellulase activities of the purified proteins**. Endoglucanase (CMCase) activity (A), Cellobiohydrolase (*p*NPCase) activity (B) and filter paper (FPase) activity (C) measured at 50-95°C. Enzyme activities of TCE1-FLE after 0-1440 min incubation at 60°C, 70°C, or 85°C (D, E and F). Enzyme activities of TCE1-RIG after 0-1440 min incubation at 60°C, 70°C, or 85°C (G, H and I). Error bars are representing the standard deviation between three independent measurements.

The purified enzymes of the transformants with the fusion gene *tce1-fle *and *tce1-rig*, actually including the intact fusion protein and the cleaved E1 from some fusion protein (Figure 4), were tested for their activity against *p*-nitrophenyl-β-D-cellobioside (*p*NPCase or cellobiohydrolase activity), carboxymethylcellulose (CMCase or endoglucanase activity) and filter paper (FPase activity). The optimal temperatures were similar for both transformant sets: 70°C for *p*NPCase activity, 85°C for CMCase activity, and 60°C for FPase activity (Figure 5). However, at the same optimal temperatures, the two fusion proteins possessed different maximum values for the three cellulase activities (Figure 5A, B, C). In fact, compared to TCE1-FLE, TCE1-RIG had significantly higher activities for *p*NPCase, CMCase and FPase at the tested temperature range (Figure 5A, B, C). For example, FPase activity of TCE1-RIG (0.95 U/mg protein) increased 70% compared to the activity of TCE1-FLE (0.55 U/mg protein) (Figure 5C). Moreover, TCE1-RIG had a better thermostability for its *p*NPCase (Figure 5H, E) and FPase activity (Figure 5I, F) at 60°C and 70°C after tracing the activities for incubation times up to 24 h at 60°C, 70°C and 85°C.

These above results demonstrated that the rigid α-helix linker was more suitable for the activity and stability of the fusion proteins. A possible explanation could be that the rigid α-helix linker provides enough space or specific physical adaptation between CBHI and E1, and therefore, maintained the high activities of the fusion protein [31,34-36]. For instance, the fusion protein with the rigid linker had a higher hydrophobicity compared to the flexible linker (TCE1-RIG had 258 AILFWV amino acid residues, while TCE1-FLE had 246 such residues). The potential for ionic interactions was also higher in the fusion protein with the rigid linker (TCE1-RIG had 134 DEKR amino acid residues, while TCE1-FLE had 126 ones). Compared to the native CBHI, the advantage of TCE1-RIG in physical mechanisms was much more significant (increased 20.73% and 11.70% in AILFWV and DEKR amino acid residues, respectively). Another possible explanation could be that the rigid α-helix linker was more stable. The *p*NPCase activity of the purified native CBHI was drastically decreased after 30 min of incubation at 60°C (data not shown). It means that both the activity and thermostability of *p*NPCase of the purified proteins depended on the intact fusion proteins (CBHI and E1 interact together). The higher *p*NPCase activity and thermostability of the purified enzymes with rigid linker (Figure 5H, E) may imply that the rigid one is more stable than the flexible one. The salt bridges of (EAAAR)_4 _present within the rigid linker were most likely involved in its higher stability [35].

### The heterogolous expression of the fusion protein had a large impact on the secreted enzymes and its ability to hydrolyze PCS

**Figure 6:**
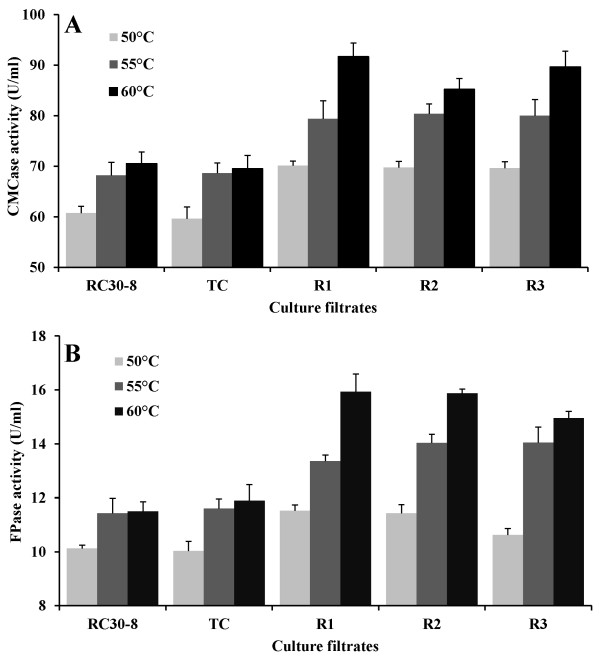
**CMCase and FPase activities of the secreted enzymes from *T. reesei *RC30-8 and its transformants**. CMCase activity of *T. reesei *RC30-8, control strain TC, and transformants R1, R2 and R3 (all containing TCE1-RIG) (A). FPase activity of reference RC30-8, control strain TC, and transformants R1, R2 and R3 (all containing TCE1-RIG) (B). Error bars are representing the standard deviation between three independent measurements.

FPase activities were measured in culture filtrates from 23 positive transformants with *tce1-rig *constructs. The FPase activities of all transformants increased significantly (*P *< 0.05) by 10-30% compared to their parent strain RC30-8 at 60°C (data not shown). R1, R2 and R3 from the tested transformants were selected for further characterization based on their high FPase activities. The activities of FPase and CMCase are normally measured at 50°C, however, the activities of RC30-8 and the transformants had their optimum at 60°C. Besides, the increase in CMCase and FPase activities were much more significant between the transformants and their reference strain at 60°C. The transformants R1, R2 and R3 had an average increase of 26% (*P *< 0.001) in CMCase activity and 36% (*P *< 0.01) in FPase activity compared to the parent strain at 60°C (Figure 6). The most efficient transformant, R1, exhibited a 30% increase in CMCase activity and a 39% increase in FPase activity.

The activities of the enzyme set within the culture filtrate from the transformant with an over-expressed CBHI (transformation control indicated as TC) were completely similar to the parent strain RC30-8 after growth on a mixture of wheat bran and cellulose. Apparently the cellobiohydrolase activity is saturated in the enzyme set of RC30-8 and, therefore, over-expression of CBHI did not result in an increase of the FPase activity. These results demonstrated that the successful expression of the fusion proteins containing the bacterial thermostable endoglucanase E1 had contributed to an increased CMCase activity in the secreted enzyme mixture. As a result of this, the total cellulase activity (measured with filter paper) was also increased in the secreted enzyme mixture of the transformants.

**Figure 7:**
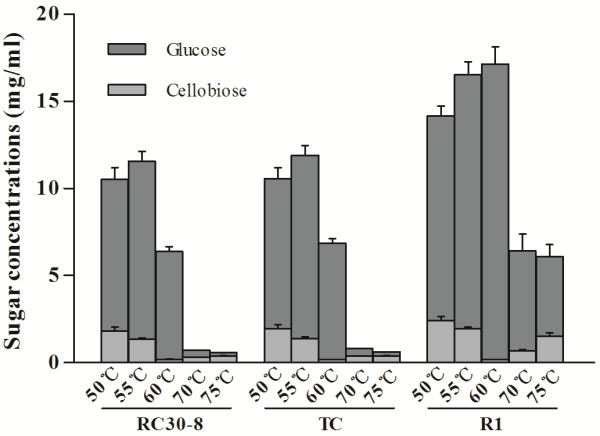
**Sugar composition (glucose and cellobiose concentrations) in the hydrolysate of PCS**. The sugar composition was analyzed by HPLC after incubating PCS with the crude secreted enzymes of *T. reesei *RC30-8 or its transformants R1, R2 and R3 (all containing TCE1-RIG) at 50-75°C. Error bars are representing the standard deviation between three independent measurements.

To further analyze the contribution of the highly-expressed fusion proteins, a saccharification experiment was performed by incubating PCS with the secreted enzyme set of *T. reesei *RC30-8 or its transformants at 50-75°C. The subsequent sugar analysis with HPLC detected only glucose and cellobiose in the hydrolysates from PCS (Figure 7). According to the total reduced sugar concentrations (meaning the sum of glucose and cellobiose), the enzyme sets of *T. reesei *RC30-8 and the transformant TC showed a similar ability to hydrolyze PCS at the different temperatures. The enzyme sets of all strains showed higher efficiency of PCS hydrolyses at 55°C than that at 50°C. However, compared to *T. reesei *RC30-8, the enzyme set of transformant R1 showed an increase in reduced sugar concentrations in the PCS hydrolysate at 55°C of 40% (*P *< 0.001).

The cellobiose concentrations after hydrolyzing PCS at 60°C decreased sharply to near zero for the secreted enzyme set of all strains (Figure 7). This observation was likely explained by a more efficient hydrolysis of cellobiose at 60°C due to the optimal temperature at approximately 60°C of the β-glucosidase enzymes in *T. reesei *[37-39]. In comparison with that at 55°C or 50°C, CMCase and FPase activities of the secreted enzyme mixture from RC30-8 and TC were higher at 60°C (Figure 6A, B), however, the total reduced sugar concentration of PCS hydrolysate at 60°C decreased significantly (Figure 7). The lower reduced sugar concentrations in the PCS hydrolysate of the reference strains at 60°C were probably explained by the different imcubation times used in the measurements of CMCase and FPase activity and the saccharification experiment. The incubation times in the measurement of *in vitro *enzyme activities were between 30 and 60 min, while the saccharification experiment lasted 24 h. Therefore, the low thermostability of the native enzymes from *T. reesei *RC30-8 and TC might lower the saccharification of PCS at 60°C. Moreover, other methodological differences such as optimal pH and substrate accessibility most likely influenced the hydrolytic efficiency [40,41]. In contrast, compared to 50°C, the reduced sugar concentration (mainly the glucose concentration) in the PCS hydrolysate of transformants R1 increased significantly at 60°C. The reduced sugar concentration resulting from transformant R1 was almost three-fold as much as that from the parent strain or TC at 60°C. At 70°C or 75°C, the enzyme set of RC30-8 or transformant TC almost completely lost the ability to hydrolyze PCS. The PCS-hydrolytic efficiency of R1 also decreased greatly at 70°C or 75°C, with the glucose concentration being about one-third of that at 60°C (Figure 7). However, the cellobiose concentration of transformant R1 increased slightly at 70°C or 75°C. This was likely due to a relative high thermostability of the fusion protein TCE1-RIG and E1 mixture at 70°C. These results demonstrated that heterologous expression of a thermostable endoglucanase *e1 *in *T. reesei *improved the overall quality of its cellulase mixture due to increased enzyme activities and a far better thermostability.

## Conclusions

The direct engineering of the *cbh1 *promoter of *T. reesei *by replacing the three binding sites of the carbon catabolite repressor CREI for the binding sites of different transcription activators showed to be a highly efficient strategy to improve the strength of a promoter. This study also demonstrated that gene fusion with a rigid linker should be a successful approach for improvement of heterologous expression efficiency of bacterial cellulases within fungi. The high activity and improved thermostability of the fusion protein in this case-study indicated that supplementing the cellulase complex of *T. reesei *with thermostable cellulases, especially in form of fusion proteins, greatly improved the ability to release sugars from lignocellulosic biomass such as PCS.

## Methods

### Microbial strains, plasmids and primers

*Escherichia coli *DH5α served as the cloning host (Novagen, Gibbstown, NJ, USA). *Agrobacterium tumefaciens *AGL1 was used as a T-DNA donor to maintain the constructs and for fungal transformation [42]. The *T. reesei *strain RC30-8, which was screened from mutants of Rut-C30 and maintained in this laboratory, was used as the host for heterologous expression. A T-DNA binary vector, pDHt/sk, containing the *hph *coding for hygromycin B phosphotransferase (under control of the *Aspergillus nidulans trpC *promoter and terminator) was used to construct the transformation vectors. The thermostable endocellulase E1 was obtained from *A. cellulolyticus *11B (ATCC^® ^Number: 43068) using primers EIGF, EIHF, EISF and EIR. Different forward primers were used to generate corresponding 5'-end to respective linker peptide coding sequences for overlapping (Table 1).

**Table 1:** Oligonucleotides used in this study

Primer	Experimental purposes	Primer sequences (5'→3')
EIGF	Overlap with cbh1GR	GGCGGCGGCGGCAGCAAGCGGGCGGGCGGCGGCTATTGG

EIHF	Overlap with cbh1HR	GAGGCCGCCGCCCGCAAGCGGGCGGGCGGCGGCTATTGG

EISF	Overlap with signal peptide region of *cbh1*	GTTGGCCGTCATCACGGCCTTCTTGGCCACAGCTCGTGCTGCGGGCGGCGGCTATTGG

EIR	Cloning of gene *e1*	GCTCTAGATCAATGATGATGATGATGATGGCCGACAGGATCGAAAATCG

pcbh1F	Cloning of *cbh1 *promoter	GGACTAGTTTTCCCTGATTCAGCGTACCCG

pcbh1R	Cloning of *cbh1 *promoter	GCTCTAGATTGACTATTGGGTTTCTGTGCC

pcbh1-1R	Site-directed mutagenesis	CGTTGCTTCTGTTTAGCCACAAGCCG

pcbh1-1F	Site-directed mutagenesis	CGGCTTGTGGCTAAACAGAAGCAACG

pcbh1-2R	Site-directed mutagenesis	CAACGGCAAAGCCAATCTTCCAATCGTTTGTTTCTTCACTCA

GFPF	Cloning of *egfp*	GCTCTAGAGTACCGGTCGCCACCATGGTG

GFPR	Cloning of *egfp*	GCTCTAGATTACTTGTACAGCTCGTCCA

cbh1F	Cloning of *cbh1*	GCTCTAGAATGTATCGGAAGTTGGCCGTC

cbh1GR	Overlap with EIGF	ACGGCGGCGGCGGCGACGGCGGCGGCGGCGACGGCGGCGGCGGCGACGGCGGCGGCGGGTCCGTGACTCTCATCATTCC

cbh1HR	Overlap with EIHF	GGGCGGCGGCCTCGCGGGCGGCGGCCTCGCGGGCGGCGGCCTCGCGGGCGGCGGCCTCCAGGCACTGAGAGTAGTAAGGG

actF	RT-PCR	TCCTTGCCTTGCGTCATCTAT

actR	RT-PCR	CACCAATCACTCTCCTGCTACAA

GFPrtF	RT-PCR	AGTGCTTCAGCCGCTACCC

GFPrtR	RT-PCR	GATGCCGTTCTTCTGCTTGTC

Note: Restriction sites are underlined

### Site-specific mutagenesis of *cbh1 *promoter

The *cbh1 *promoter was amplified from *T. reesei *RC30-8 genomic DNA with the primers pcbh1F and pcbh1R (Table 1), which was designed according to the published sequence (http://genome.jgi-psf.org/Trire2/Trire2.home.html). To mutate the CREI-binding element located at -724 (5'-GTGGGG-3') in the *cbh1 *promoter, an overlap PCR was carried out with primers pcbh1F, pcbh1-1R, pcbh1-1F, and cbh1R. The generated fragment with the artificial ACEII-binding site named pcbh1m1 (Figure 1) was manipulated with another overlap PCR to mutate the remaining two CREI-binding elements (complementary sequence of 5'-GTGGGG-3' located at -698 and -690) using primers pcbh1F, pcbh1-2R, pcbh1-2F, and cbh1R in a similar way. Finally, the promoter after two rounds of mutagenesis was renamed pcbh1m2 (Figure 1) in which the ACEII-binding site substituted for the CREI-binding element located at -724, and two CCAAT boxes replaced the other two CREI-binding elements.

### The construction and transformation of expression vectors for heterologous genes (*egfp*, *e1*) and the fusion genes of *cbh1 *and *e1*

The *cbh1 *promoter and its mutated fragments were double digested with SpeI and XbaI, and ligated into the SpeI/XbaI site of plasmid pDHt/sk to obtain vectors pDHt/sk-pcbh1, pDHt/sk-pcbh1m1, pDHt/sk-pcbh1m2. The reporter gene *egfp *was then introduced into the XbaI site of all four vectors after a PCR carried out with primers GFPF and GFPR and the *egfp *expression vectors were constructed.

The catalytic domain fragment of *e1 *(GenBank: U33212.1) was fused with the intact *cbh1 *coding region via a flexible neutral polyglycine linker (GGGGS)_4 _and a rigid α-helix linker (EAAAR)_4_, using overlapping primers cbh1GR and cbh1HR (Table 1). The generated fragments were named *tce1-fle *and *tce1-rig*. The catalytic domain of *e1 *was fused with the signal sequence of *cbh1 *using the primers cbh1F and E1R and named *se1h*. All fragments were also introduced into a 6 × His-tag coding region at its 3'-end. The original *e1 *fused with a his-tag was named *e1h*. After ligated into the XbaI site of plasmid pDHt/sk-pcbhm2, the heterologous expression vectors harboring *tce1-fle*, *tce1-rig, se1 *and *e1h *were constructed. The *cbh1 *fragment was also ligated into the same vector for transformation control. The construction of the fusion genes is shown in Figure 3.

All of the expression vectors for the heterologous genes were transformed into the recipient *T. reesei *RC30-8 using *Agrobacterium*-mediated fungal transformation [43].

### Selection and culture of transformants or *T. reesei *RC30-8

Transformants were selected using hygromycin B (10 μg/ml) and cefotaxime (300 μM) on potato dextrose agar (PDA). Each positive transformant was used to create monoconidial cultures for genetic stability and confirmed single-copy integration of *egfp *using real-time PCR. All fungal strains including *T. reesei *RC30-8 were spread on PDA plates and were grown at 28°C for about 7 days and then stored at 4°C after conidia formed. The conidia of the fungal transformants were collected from PDA plates and inoculated into 50 ml flasks containing 10 ml Sabouraud dextrose broth (SDB) and cultured for 2 days at 28°C and 200 rpm on a rotary shaker for protein expression. Subsequently, 1 ml of the culture was transferred to flasks with 10 ml minimal medium plus different carbon resources [3% cellulose powder (CF-11, Whatman, Maidstone, England) and 2% wheat bran (ground to less than 0.5 mm in diameter by a mill at the lab) as inducer or 2% glucose as repressor] and incubated at 28°C and 200 rpm. The minimal medium contained 0.4% KH_2_PO_4_, 0.28% (NH_4_)_2_SO_4_, 0.06% MgSO_4_·7H_2_O, 0.05% CaCl_2_, 0.06% urea, 0.3% tryptone, 0.1% Tween 80, 0.5% CaCO_3_, 0.001% FeSO_4_·7H_2_O, 0.00032% MnSO_4_·H_2_O, 0.00028% ZnSO_4_·7H_2_O, 0.0004% CoCl_2 _[44] and was adjusted to pH 5.5.

### Qualitative and quantitative evaluation of promoter strength

After being cultured for 2 days, mycelia of transformants were collected for fluorescence observation or for total RNA extraction, after rinsing with sterilized water three times. The FastPrep^®^-24 (MP Biomedicals, Solon, HO, US) instrument in combination with TRIzol^® ^Reagent (Invitrogen, Carlsbad, CA, USA) was successfully used for total RNA extraction. Reverse transcription was carried out using the PrimeScript^® ^RT reagent Kit (Takara, Dalian, China). Relative expression levels of *egfp *were calculated in comparison with the expression of *act *encoding actin by RT-PCR with primers GFPrtF, GFPrtR, actF and actR.

### Purification of fused proteins from the *T. reesei *transformants

After being cultured for 7 days, the culture filtrate of transformants was collected by a centrifugation at 4°C and 8,000 × *g *for 10 min. The fusion proteins TCE1-FLE or TCE1-RIG in the collected culture filtrate were purified using Novagen Ni-NTA His•Bind^® ^Resin (Merck, Darmstadt, Germany) with step gradient elution. The 40 mM imidazole washouts were collected, and the buffer was exchanged with 20 mM pH 7.4 NaH_2_PO_4 _using a Vivaspin™ ultrafilter (10 kDa cut-off, GE Healthcare Piscataway, NJ, USA) to remove the imidazole. Fusion protein production was examined by SDS-PAGE and Western blotting using an anti-His antibody (Yeli, Shanghai, China). Proteins were quantified using the DC protein assay kit (Bio-Rad, Hercules, CA, USA), according to the manufacturer's instructions.

### Enzyme activity assays

The crude secreted enzymes of fungal strains (the culture filtrate of *T. reesei *RC30-8 or its transformants was collected by a centrifugation at 4°C and 8,000 × *g *for 10 min after being cultured for 7 days) or the purified TCE1-FLE and TCE1-RIG were used to examine substrate specificity and to characterize its properties. CMCase activity was assayed by measuring the amount of reducing sugar released from CMC (Sigma, St. Louis, MO, USA) using the DNS method [45]. The assay mixture contained a specific amount of diluted enzyme, 100 μl of 2% CMC and 100 μl 50 mM saline sodium citrate buffer (SSC, pH 5.0). The mixture was incubated at 60°C for 10 min; 200 μl DNS was added to stop the reaction, followed by incubation for 5 min in boiling water. Photometric assays were analysed at OD_540 _using a Varioskan Flash microplate reader (ThermoScientific, Rockford, IL, USA).

Cellobiohydrolase activity was measured as reported by Deshpande [46] with slight modifications. In brief, 90 μl of 4 mM *p*NPC solution (50 mM SSC, pH 5.0) containing 1 mg/ml D-glucono-1,5-*σ*-lactone was incubated with 20 μl of diluted culture supernatant at 50°C for 30 min. Then, 100 μl of each sample was mixed with 100 μl 2% sodium carbonate, and photometric assays were carried out at OD_400_.

The specific activities of the crude enzymes secreted by *T. reesei *RC30-8 or its transformants or the purified fusion proteins on FPase activity were measured using a modified IUPAC method [47]. The assay mixture was incubated at an optimal condition of 60°C, pH 5.0 for 60 min, and the reaction was stopped with 120 μl DNS followed by an incubation in boiling water for 10 min. A unit of enzyme activity (U) was defined as the number of micromoles of reducing sugar or *p*NP released per minute per milligram protein or per milliliter fermented culture. Student's *t*-test was performed with Excel 2007 (Microsoft, WA, USA), employing a two-tailed test.

The thermostability of the purified proteins or the secreted crude enzymes was assayed by the similar enzyme activity assay methods mentioned above. Samples were exposed to thermal stress in water baths at 60°C, 70°C and 85°C for up to 24 h. Three aliquots were pipetted out for enzyme activity assays at intervals of 15 min-12 h during the exposure.

### Hydrolysis assay of PCS

In the hydrolysis of dilute sulfuric acid pretreated corn stover [48], using the culture filtrate of *T. reesei *RC30-8 or its transformants as crude enzyme preparations, 3% PCS (30 mg) and 30 μl different enzyme preparations were incubated in 50 mM saline sodium citrate (pH 5.0) at 50°C, 55°C, 60°C, 70°C and 75°C for 24 h, and the total volume of the saccharification mixture was 1 ml. The hydrolysates were then centrifuged at 4°C, 12000 × *g *for 20 min and the concentrations of glucose and cellooligosaccharides in the supernatant were measured by high-performance liquid chromatography (HPLC; LC-20A; Shimadzu, Kyoto, Japan), using a refractive index detector (Shimadzu, Kyoto, Japan). The hydrolysates were filtered using a 0.22 μm pore size membrane (Millipore Corporation, Billerica, MA) and then separated on a Shodex Asahipak NH2P-50 4E column (Showa Denko K.K., Kanagawa, Japan) with isocratic elution (75% acetonitrile, 25% H_2_O [vol/vol]) at 1 ml/min.

## Competing interests

The authors declare that they have no competing interests.

## Authors' contributions

GZ performed main experiments and data analysis, and drafted the manuscript. SS performed the modification of promoters. YJ participated in the expression of e1 directly in *T. reesei*. JB and RV commented on and polished the manuscript. JZ carried out enzyme assays. LC and LM participated in transformation. ZZ and CW supervised the study and revised the manuscript. All authors read and approved the manuscript.
